# An Externally-Applied, Natural-Mineral-Based Novel Nanomaterial IFMC Improves Cardiopulmonary Function under Aerobic Exercise

**DOI:** 10.3390/nano12060980

**Published:** 2022-03-16

**Authors:** Tomohiro Akiyama, Shinnosuke Hatakeyama, Kazuhisa Kawamoto, Hideko Nihei, Takamichi Hirata, Tomohiro Nomura

**Affiliations:** 1 Advanced Research Laboratories, Tokyo City University, Tokyo 158-0082, Japan; thirata@tcu.ac.jp; 2 Graduate School of Education, Kyoto University, Kyoto 606-8501, Japan; 3 Graduate School of Global Environmental Studies, Sophia University, Tokyo 102-8554, Japan; 4 School of Integrative and Global Majors, University of Tsukuba, Tsukuba 305-8577, Japan; 5 Graduate School of Information Technology, Kobe Institute of Computing, Kobe 650-0001, Japan; 6 School of Social and Behavioral Sciences, Nanjing University, Nanjing 210023, China; 7 Institute for Humanities in Africa, University of Cape Town, Rondebosch 7701, South Africa; 8 Graduate School of Integrative Science and Engineering, Electrical Engineering and Chemistry, Tokyo City University, Tokyo 158-8557, Japan; g1981258@tcu.ac.jp; 9 Graduate School of Human Development and Culture, Fukushima University, Fukushima 960-1296, Japan; kawamoto@educ.fukushima-u.ac.jp; 10 Sports Division, Fukushima Prefecture, Fukushima 960-8065, Japan; nihei@sports-fukushima.or.jp; 11 Osaka City University, Osaka 558-8585, Japan; t.nomura@abeam.ocn.ne.jp

**Keywords:** nanotechonology, natural-mineral-based novel nanomaterial, nitric oxide, hemoglobin, heartrate, cardiopulmonary function improvement

## Abstract

Nanotechnology has widespread applications in sports; however, there are very few studies reporting the use of nanotechnology to enhance physical performance. We hypothesize that a natural-mineral-based novel nanomaterial, which was developed from Japanese hot springs, might overcome the limitations. We examined if it could enhance physical performance. We conducted a treadmill exercise test on 18 students of athletic clubs at Fukushima University, Japan, and measured heart rate, oxygen consumption, maximal oxygen consumption, CO${}_{2}$ production, and respiratory quotient 106 times in total. The results showed that the elevation of heart rate was significantly suppressed in the natural-mineral-based nanomaterial group, while no differences were observed in oxygen consumption, maximal oxygen consumption, CO${}_{2}$ production, and respiratory quotient between groups. To our knowledge, this result is the first evidence where an improvement of cardiovascular and pulmonary functions was induced by bringing a natural-mineral-based nanomaterial into contact with or close to a living body without pharmacological intervention or physical intervention. This could open new avenue of biomedical industries even in an eco-friendly direction. The precise mechanisms remain a matter for further investigation; however, we may assume that endothelial NO synthase, hemoglobin and endothelium-derived hyperpolarizing factor are deeply involved in the improvement of cardiovascular and pulmonary functions.

## 1. Introduction

Nanotechnology, which functions at a scale smaller than 100 nm (1 nm = 10${}^{9}$ m), has the potential for groundbreaking technological innovation in various fields. The National Nanotechnology Initiative (NNI) was established in the U.S. in 2000, signifying the importance of nanotechnology as a promising area of research and a national priority [1]. Since the NNI was established, similar programs have been established in more than 60 countries, and nanotechnology research has advanced rapidly [1]. Nanotechnology is expected to have applications across a variety of areas such as electronics, mechatronics, chemistry, energy, biology, environment, midicine, healthcare, and sports.

In the field of athletics, nanotechnology has already been widely used for athletic equipment, sports coating, clothes, devices, and supplements [2]. Sports equipment utilizing nanomaterials is lightweight and achieves higher performance and improved durability. Carbon nanotubes have been applied to tennis rackets and badminton rackets to improve elasticity, durability, and texture while reducing weight [3,4]. Functional sportswear made of nanofibers has improved water absorbability, sorbability, and air permeability [2,5]. Nanotechnology has enabled products such as antidromic, impermeable, and antibiotic insoles, impermeable swimwear, and sports shoes that are highly resistant to shock [2,6]. Although nanotechnology has widespread applications in sports, there are very few studies reporting the use of nanotechnology to enhance physical performance. One of these studies reported that nanosilica particles with a diameter of 70 nm evoked vascular relaxation; however, these particles have to be taken into the body rather than applied to the skin externally [7]. Because nanotechnology also has potential toxicity [8,9], human applications require close attention to safety.

In the meantime, modern medicine has been very successful at improving cardiovascular and pulmonary functions. However, cardiovascular and pulmonary diseases are still the leading causes of morbidity and mortality worldwide. In this avenue, recent progress on nanomaterials opened up new opportunities for improve cardiovascular and pulmonary functions [10,11,12,13,14]. The special properties of nanomaterials such as their intrinsic physiochemical properties, surface energy, and surface topographies could actively enhance desirable cellular responses within the cardiovascular system, projecting a growing potential for clinical translation [12]. Polizzi et al. [15] fabricated monolayer-protected gold clusters (MPCs) capable of releasing nitric oxide (NO), facilitating many physiological processes, and supporting the healthy function of the cardiovascular system [16]. However, these require introducing nanomaterials into the human body, and the use of nanomaterials has become controversial due to health risks associated with their applications [17].

The natural-mineral-based novel nanomaterial, which was developed from Japanese hot springs and named Integrated Functional Mineral Crystal (IFMC) by Teikoku Pharmaceutical Co., Ltd., Osaka, Japan [18], may overcome the limitations in previous studies. Advantages of the IFMC are four-fold: (1) it is composed of naturally derived substances and does not contain any toxic or hazardous substances or elements, (2) it is easy to use (just putting it or spraying it in a solution on the body), (3) it requires neither its intake into the body nor absorption through the skin, and (4) it is inexpensive. The results of a preliminary experiment showed that blood circulation and sense of equilibrium improved by bringing the IFMC into contact with or close to a living body without pharmacological intervention or physical intervention [19,20]. It is patented as an external material applied to the skin to promote blood circulation and improve balance [20]. Since blood circulation can be promoted by the IFMC, cardiopulmonary function under aerobic exercise may also be improved. If it were able to enhance physical performance with only external contact to human skin, it is expected to have widespread applications in the fields of medicine, healthcare, and sports.

In this study, we examined the hypothesis that IFMC enhance physical performance with only external contact to human skin using a treadmill exercise test in student members of athletic clubs at Fukushima University, Japan. In this experiment, heart rate, oxygen consumption, maximal oxygen consumption, carbon dioxide (CO${}_{2}$) production, and respiratory quotient were measured in students with and without a T-shirt impregnated with the IFMC. The rest of the article is composed of three sections: the Materials and Methods and the Results of this experiment, and the Discussion of the results are described in Section 2, Section 3 and Section 4, respectively.

## 2. Materials and Methods

**Figure 1:**
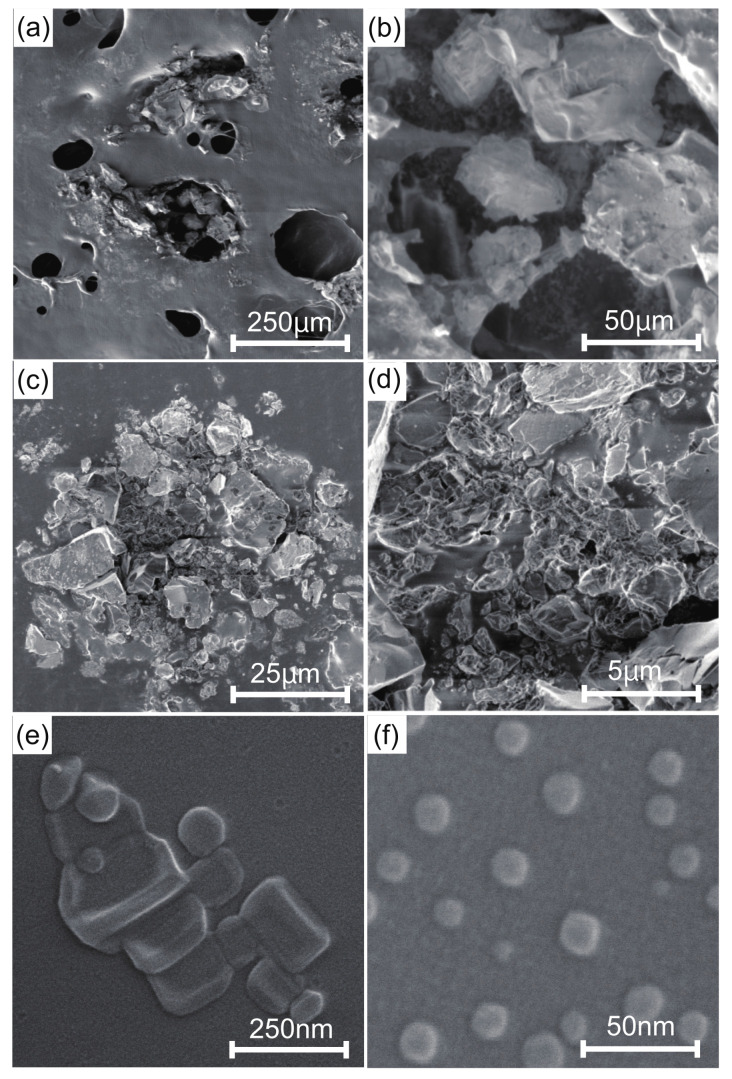
FE-SEM images of IFMC [19]: (**a**) ×100 at 1 kV, (**b**) ×500 at 1 kV, (**c**) ×1000 at 1 kV, (**d**) ×5000 at 1 kV, (**e**) ×100,000 at 10 kV, and (**f**) ×200,000 at 10 kV. Reprinted from Reference [19].

**Figure 2:**
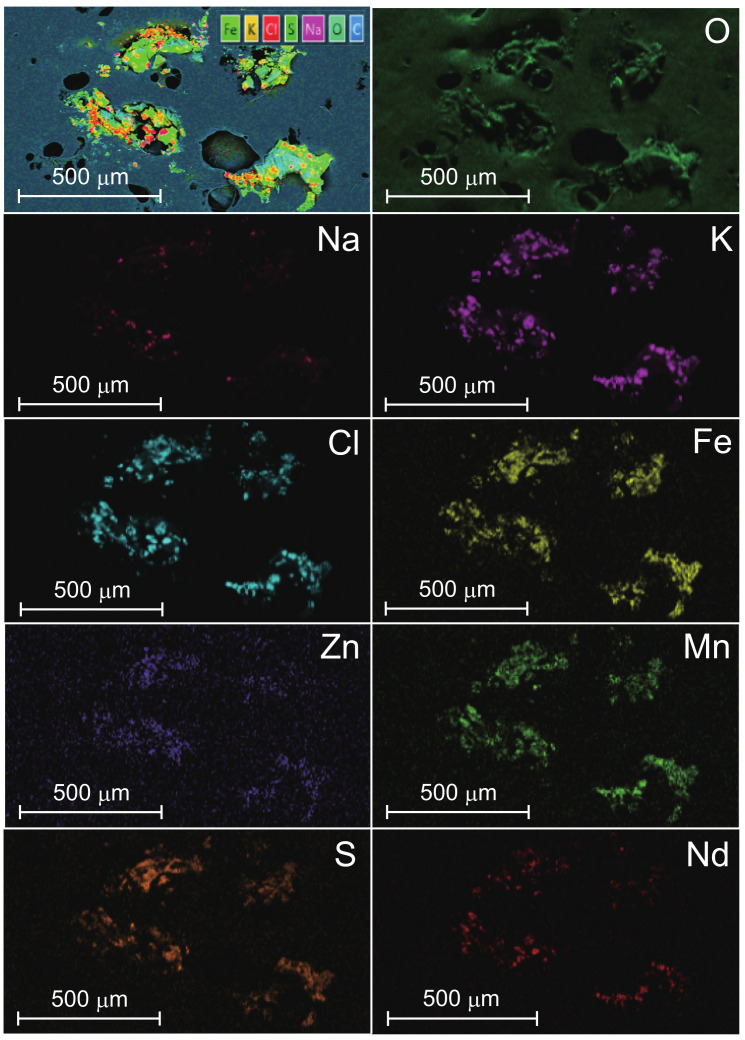
EDS element mapping corresponding to the SEM image of Figure 1a [19]. Elements include oxygen (O), sodium (Na), sulphur (S), potassium (K), chlorine (Cl), manganese (Mn), iron (Fe), zinc (Zn), and neodymium (Nd). Reprinted from Reference [19].

The experimental object of this research is the natural-mineral-based novel nanomaterial IFMC by Teikoku Pharmaceutical Co., Ltd., Osaka, Japan [20]. The IFMC is produced focusing on spa therapy by immersion in iron-rich hot spring water for a certain period to combine it with several minerals. A solution of IFMC consists of haematite (Fe${}_{2}$O${}_{3}$), olivine (Mg${}_{2}$SiO${}_{4}$ and Fe${}_{2}$SiO${}_{4}$), rhodolite (MnCO${}_{3}$), zincite (ZnCO${}_{3}$), and additives such as deionized water, ethanol, methylparaben and sodium metabisulphite. Figure 1 shows SEM images of IFMC [19]. Akiyama et al. observed different sizes and forms of crystals, including nano-sized crystals. Figure 2 shows element mapping images [19]. The distribution of sulphur (S), manganese (Mn), iron (Fe), zinc (Zn), and neodymium (Nd) originating in mineral components extracted from natural minerals was confirmed. The concentration in descending order was as follows: O, Cl, K, Na, Fe, Zn, Mn, S, and Nd.

The subjects of this research included eight male and ten female students who belong to athletic clubs at Fukushima University, Japan. Table 1 shows the age and physical characteristics of each subject. The objectives, methods, and safety of this study were fully explained to subjects in advance, and informed consent was obtained from each subject. The experimental human protocol was reviewed and approved by the Research Ethics Committee of Fukushima University, Japan (Approval No. 29-18). This experiment was a double-blind, placebo-controlled study. In this experiment, subjects in the IFMC group were instructed to wear the IFMC-impregnated T-shirt, while subjects in the placebo group were instructed to wear a T-shirt with the same material and appearance but without IFMC. Students who participated in this experiment were randomly assigned into the IFMC group (n = 49) or the placebo group (n = 51). The IFMC group included 24 male subjects and 25 female subjects. The placebo group included 26 male subjects and 25 female subjects.

In this experiment, a treadmill (Power JOG JX200, Cardiosport, Waterlooville, UK) was set to a gradient of 0%. Taking into account that the fluctuation in physiological response depends on the ambient temperature, the temperature of the experiment room was adjusted to 25 °C to 26 °C using an air conditioner. Moreover, the temperature, humidity, CO${}_{2}$, and atmosphere of the experiment room were controlled in real-time using a self-build sensor. Before starting the experiment, the subjects were generally instructed to run at a heart rate of 140 bpm and their oxygen consumption became steady [21,22,23,24]. We followed the procedure, which is important to avoid the effect of the heart rate decrease due to improvement of stroke volume during submaximal exercise. Through this warming up, the treadmill speed at which each subject can run at a heart rate of 140 bpm was identified. Heart rate, oxygen consumption, maximal oxygen consumption, CO${}_{2}$ production, and respiratory quotient were continuously measured throughout the warm-up as well as the following 20 min of running. For exhalation-gas analysis, a pulmonary monitoring system (AE-310SRC, Minato Medical Science Co., Ltd., Osaka, Japan) and a mask (AE-310SRC, Minato Medical Science Co., Ltd., Osaka, Japan) were used. A heart rate sensor (A300, Polar Electro, Kempele, Finland) was also used to measure heart rate.

The data were analyzed starting from 20 min after the subjects’ oxygen consumption became steady. Based on the results for each parameter, the mean heart rate, oxygen consumption, maximal oxygen consumption, CO${}_{2}$ production, and respiratory quotient, as well as the standard error of the sample mean, were calculated for the IFMC group and the placebo group. A paired *t*-test was used for statistical comparison. For each test, the significance level was set to less than 5%. In the process of data screening, five measurements and one measurement were excluded because of incorrect measurement due to displacement of the measuring instrument and out of the normal distribution, respectively.

## 3. Results

### 3.1. Heart Rate

**Figure 3:**
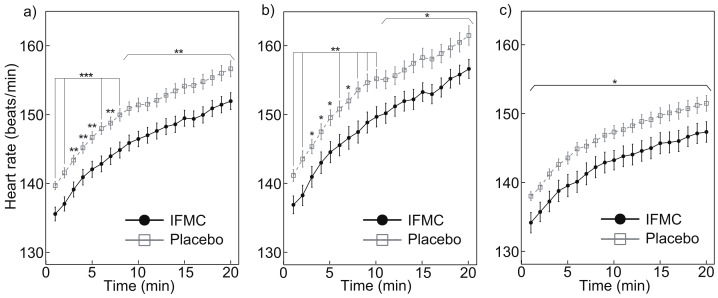
Differences in (**a**) all, (**b**) male, and (**c**) female heart rate over 20 min between IFMC and placebo applications. The symbols *, **, and *** stand for significant difference between IFMC and Placebo groups at *p* < 0.05, *p* < 0.01, and *p* < 0.001, respectively.

Figure 3 shows the measured heart rates. Figure 3a shows a comparison between of heart rate between the IFMC group and the placebo group. In the placebo group, the mean heart rate was 139.7 bpm at 1 min, 156.7 bpm at 20 min, and 150.3 bpm over the 20 min period. In the IFMC group, the mean heart rate was 135.6 bpm at 1 min, 152.0 bpm at 20 min, and 145.7 bpm over the 20 min period. The mean heart rate in IFMC group was statistically significantly lower for all time points during the 20 min experiment.

Figure 3b,c show the measured heart rate in male and female subjects, respectively. The mean heart rate in male subjects in the placebo group was 141.2 bpm at 1 min, 161.6 bpm at 20 min, and 153.8 bpm over the 20 min period. The mean heart rate in male subjects in the IFMC group was 137.0 bpm at 1 min, 156.7 bpm at 20 min, and 148.9 bpm over the 20 min period. The mean heart rate in the female subjects in the placebo group was 138.1 bpm at 1 min, 151.5 bpm at 20 min, and 146.7 bpm over the 20 min period. The mean heart rate in female subjects in the IFMC group was 134.2 bpm at 1 min, 147.4 bpm at 20 min, and 142.6 bpm over the 20 min period. In both male and female subjects, a significant difference was observed between groups at all time points during the 20 min experiment.

### 3.2. Oxygen Consumption

**Figure 4:**
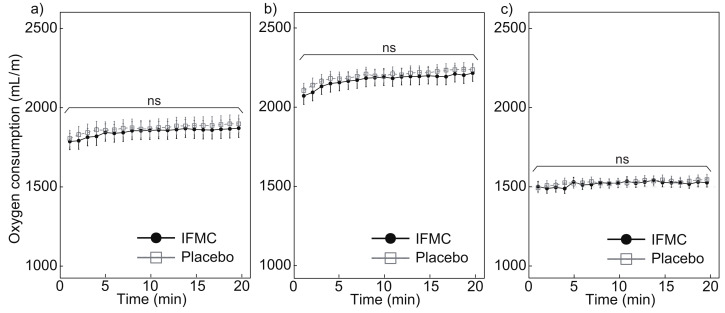
Differences in (**a**) all, (**b**) male, and (**c**) female oxygen consumption over 20 min between IFMC and placebo applications. The “ns” stands for “not significant (*p* > 0.05)”.

Figure 4 shows the oxygen consumption measured during the experiment. Figure 4a shows a comparison of oxygen consumption between the IFMC group and the placebo group. In the placebo group, the mean oxygen consumption was 1808.1 mL min${}^{-1}$ at 1 min, 1900.7 mL min${}^{-1}$ at 20 min, and 1872.8 bpm over the 20 min period. In the IFMC group, the mean oxygen consumption was 1789.3 mL min${}^{-1}$ at 1 min, 1873.4 mL min${}^{-1}$ at 20 min, and 1849.1 bpm over the 20 min period. No statistically significant difference was observed between groups for any time point during the 20 min experiment.

Figure 4b,c show the measured oxygen consumption in male and female subjects, respectively. The mean oxygen consumption in male subjects in the placebo group was 2108.9 mL min${}^{-1}$ at 1 min, 2240.3 mL min${}^{-1}$ at 20 min, and 2202.9 mL min${}^{-1}$ over the 20 min period. The mean oxygen consumption in male subjects in the IFMC group was 2075.0 mL min${}^{-1}$ at 1 min, 2218.7 mL min${}^{-1}$ at 20 min, and 2177.0 mL min${}^{-1}$ over the 20 min period. The mean oxygen consumption in female subjects in the placebo group was 1495.2 mL min${}^{-1}$ at 1 min, 1547.4 mL min${}^{-1}$ at 20 min, and 1529.4 mL min${}^{-1}$ over the 20 min period. The mean oxygen consumption in the female subjects in the IFMC group was 1503.6 mL min${}^{-1}$ at 1 min, 1528.1 mL min${}^{-1}$ at 20 min, and 1521.1 mL min${}^{-1}$ over the 20 min period. No statistically significant difference was observed between groups for any time point during the 20 min experiment.

### 3.3. Maximal Oxygen Consumption

**Figure 5:**
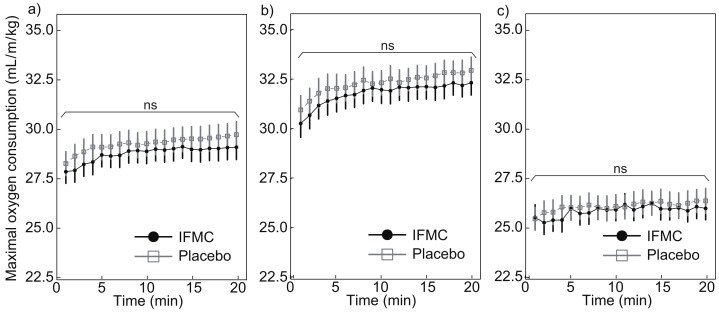
Differences in (**a**) all, (**b**) male, and (**c**) female maximal oxygen consumption over 20 min between IFMC and placebo applications. The “ns” stands for “not significant (*p* > 0.05)”.

Figure 5 shows the maximal oxygen consumption measured during the experiment. Figure 5a shows a comparison of the maximal oxygen consumption between the IFMC group and the placebo group. The mean maximal oxygen consumption in the placebo group was 28.3 mL min${}^{-1}$/kg at 1 min, 29.7 mL min${}^{-1}$/kg at 20 min, and 29.3 mL min${}^{-1}$/kg for the 20 min period. The mean maximal oxygen consumption in the IFMC group was 27.9 mL min${}^{-1}$/kg at 1 min, 29.1 at 20 min, and 28.8 mL min${}^{-1}$/kg over the 20 min period. No statistically significant difference was observed between groups for any time point during the 20 min experiment.

Figure 5b,c show the maximal oxygen consumption in male and female subjects, respectively. The mean maximal oxygen consumption in the male subjects in the placebo group was 31.0 mL min${}^{-1}$/kg at 1 min, 33.0 mL min${}^{-1}$/kg at 20 min, and 32.3 mL min${}^{-1}$/kg over the 20 min period. The mean maximal oxygen consumption in the male subjects in the IFMC group was 30.3 mL min${}^{-1}$/kg at 1 min, 32.4 mL min${}^{-1}$/kg at 20 min, and 31.8 mL min${}^{-1}$/kg over the 20 min period. The mean maximal oxygen consumption in the female subjects in the placebo group was 25.4 mL min${}^{-1}$/kg at 1 min, 26.4 mL min${}^{-1}$/kg at 20 min, and 26.1 mL min${}^{-1}$/kg over the 20 min period. The mean maximal oxygen consumption in the female subjects in the IFMC group was 25.5 mL min${}^{-1}$/kg at 1 min, 26.0 mL min${}^{-1}$/kg at 20 min, and 25.8 mL min${}^{-1}$/kg over the 20 min period. In both male and female subjects, no statistically significant difference was observed between groups at any time point during the 20 min experiment.

### 3.4. CO_2_ Production

**Figure 6:**
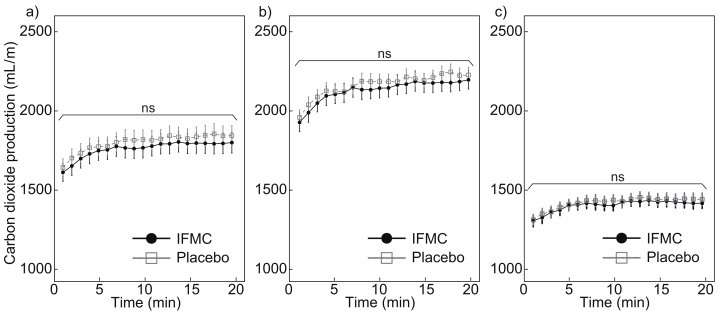
Differences in (**a**) all, (**b**) male, and (**c**) female CO${}_{2}$ production over 20 min between IFMC and placebo applications. The “ns” stands for “not significant (*p* > 0.05)”.

Figure 6 shows the CO_2_ production measured during the experiment. Figure 6a shows a comparison of CO_2_ production between the IFMC group and the placebo group. The mean CO_2_ production in the placebo group was 1643.4 mL min${}^{-1}$ at 1 min, 1843.7 mL min${}^{-1}$ at 20 min, and 1801.1 mL min${}^{-1}$ over the 20 min period. The mean CO${}_{2}$ production in the IFMC group was 1612.3 mL min${}^{-1}$ at 1 min, 1800.0 mL min${}^{-1}$ at 20 min, and 1760.4 mL min${}^{-1}$ over the 20 min period. No statistically significant difference was observed between the groups for any time point during the 20 min experiment.

Figure 6b,c show the CO${}_{2}$ production in male and female subjects, respectively. The mean CO${}_{2}$ production in male subjects in the placebo group was 1963.7 mL min${}^{-1}$ at 1 min, 2232.4 mL min${}^{-1}$ at 20 min, and 2169.8 mL min${}^{-1}$ over the 20 min period. The mean CO${}_{2}$ production in male subjects in the IFMC group was 1933.1 mL min${}^{-1}$, 2201.6 mL min${}^{-1}$ at 20 min, and 2135.2 mL min${}^{-1}$ over the 20 min period. The mean CO${}_{2}$ production in female subjects in the placebo group was 1310.4 mL min${}^{-1}$ at 1 min, 1439.5 mL min${}^{-1}$ at 20 min, and 1417.7 mL min${}^{-1}$ over the 20 min period. The mean CO${}_{2}$ production in female subjects in the IFMC group was 1304.3 mL min${}^{-1}$ at 1 min, 1414.5 mL min${}^{-1}$ at 20 min, and 1400.7 mL min${}^{-1}$ over the 20 min period. In both the male and female subjects, no statistically significant difference was observed between groups for any time point during the 20 min experiment.

### 3.5. Respiratory Quotient

**Figure 7:**
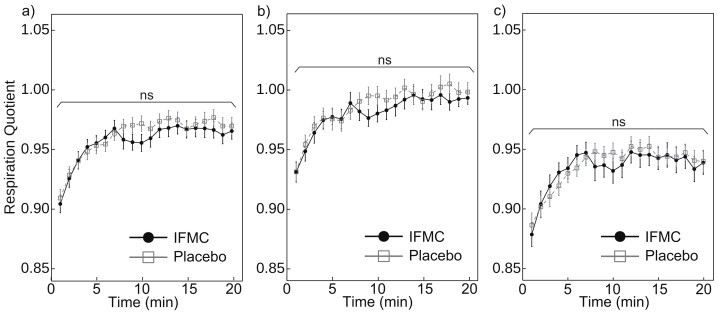
Differences in (**a**) all, (**b**) male, and (**c**) female respiratory quotient over 20 min between IFMC and placebo applications. The “ns” stands for “not significant (*p* > 0.05)”.

Figure 7 shows the respiratory quotient measured during the experiment. Figure 7a shows a comparison of respiratory quotient between the IFMC group and the placebo group. The mean respiratory quotient in the placebo group was 0.91 at 1 min, 0.97 at 20 min, and 0.96 over the 20 min period. The mean respiratory quotient in the IFMC group was 0.90 at 1 min, 0.97 at 20 min, and 0.96 over the 20 min period. No statistically significant difference was observed between groups for any time point during the experiment.

Figure 7b,c show the mean respiratory quotient in male and female subjects, respectively. The respiratory quotient in male subjects in the placebo group was 0.93 at 1 min, 1.00 at 20 min, and 0.99 over the 20 min period. The mean respiratory quotient in male subjects in the IFMC group was 0.93 at 1 min, 0.99 at 20 min, and 0.98 over the 20 min period. The mean respiratory quotient in female subjects in the placebo group was 0.89 at 1 min, 0.94 at 20 min, and 0.94 over the 20 min period. The mean respiratory quotient in female subjects in the IFMC group was 0.88 at 1 min, 0.94 at 20 min, and 0.93 over the 20 min period. In both male and female subjects, no statistically significant difference was observed between groups for any time point during the 20 min experiment.

## 4. Discussion

The results of this study support the hypothesis that IFMC enhance physical performance. Although no significant difference was observed between groups in mean oxygen consumption (Figure 4), maximal oxygen consumption (Figure 5), CO${}_{2}$ production (Figure 6), and respiratory quotient (Figure 7), a significant difference was observed in heart rate (Figure 3). The no significant differences in oxygen consumption and maximal oxygen consumption (Figure 4 and Figure 5) indicate that there was no difference in oxygen consumption between the IFMC group and the placebo group. The no significant difference in CO${}_{2}$ production (Figure 6) indicate that there was no difference in CO${}_{2}$ production between the IFMC group and the placebo group. Although the respiratory quotient was observed less than 1.0 (Figure 7) the present experiment is not to test running intensity. Thus, the suppression of heart rate elevation in the IFMC group (Figure 3) indicates that the IFMC can improve cardiopulmonary function and exercise efficiency. This would enable people to enhance their physical performance while reducing the burden on the heart. Furthermore, the relatively low mean heart rates with the use of the IFMC (Figure 3) suggest that the subjects may feel like they can run with less effort and have improved autonomic balance.

A reasonable possibility of the mechanism for the low heart rate in the IFMC group, despite having almost the same oxygen consumption volume, is that the IFMC increased the subjects’ blood flow. Akiyama et al. conducted in vivo experiments and demonstrated the IFMC can induce an increase of intravascular nitric oxide (NO), vasodilation, and the consequent increase in blood flow by bringing IFMC into contact with or close to a living body without pharmacological intervention or physical intervention [19]. Increased blood flow elevates the volume of oxygen that can be transported.

It is well known that NO is associated with increase in blood flow. NO is reported to be an endothelium-derived relaxing factor [25]. NO is produced in the body and activates guanylate cyclase through the smooth muscle cell membrane to promote the production of cystic guanosine monophosphate. As a result, the vascular smooth muscle is relaxed, the vessels are dilated, and blood flow increases [16,26]. Therefore, the significant reduction of heart rate while wearing a cloth embedded with IFMC (Figure 3) may be linked to NO.

The mechanism of NO expression caused by the use of IFMC is unknown; however, there are two reasonable possibilities [19]. The first one is NO synthesis. NO is synthesized from L-arginine by the enzyme nitric oxide synthase in the body [16,26]. However, the biological reaction via a receptor includes a time lag [27,28]. The NO level in blood increases about only 30 s after contact with IFMC [19]. Therefore, NO expression due to IFMC is not a biological receptor response, but rather expressed through an entirely different mechanism such as a physical reaction.

The second possibility involves hemoglobin (Hb). It was previously thought that NO is a gaseous signaling substance that diffuses freely between vascular endothelial cells and vascular smooth muscle cells. However, Straub et al. reported that NO does not move freely between vascular endothelial cells and vascular smooth muscle cells [29]. Straub et al. also reported that Hb present in both types of cells controls NO diffusion. Hb is made up of globin protein bound to four iron porphyrin complexes (heme iron). Because Hb binds to oxygen molecules, it is deeply involved in controlling NO signaling in addition to transporting oxygen (O${}_{2}$). It is known that iron atom displacement in Hb facilitates the signaling of O${}_{2}$ and NO in blood. The iron ion of heme iron has five-fold coordination and a high spin; however, it becomes a six-fold coordination and low spin when an oxygen molecule is coordinated. Changing the spin of a d-electron from high to low causes the iron ion to get sucked into the porphyrin ring. This change at the molecular level transforms the entire structure of Hb to increase the affinity (sorbability) of the other three heme irons to an oxygen molecule. In other words, the structure of the d-electron controls the uptake and release of oxygen molecules. This mechanism supports the hypothesis that contact with an IFMC promotes the disbanding of NO from Hb.

To clarify this hypothesis, magnetic evaluation using muon as a magnetic probe may be effective. The mechanism between IFMC and Hb may be clarified by examining how the spin transition change of an iron atom changes the affinity (sorbability) of heme iron in Hb for oxygen molecules or NO. The spin characteristics of IFMC could be observed using muon as a magnetic probe. In the spintronics area of semiconductor engineering, a study reported that simple antiferromagnetic insulators convey spin information over distances of more than tens of micrometers [30]. Spin information has not yet been conveyed over the distances found in this study; therefore, if it can be demonstrated, it would be the world’s first study.

## 5. Conclusions

The present study suggests that IFMC significantly improved cardiopulmonary function under aerobic exercise. There are very few studies of enhanced physical performance using nanotechnology, and its safety is still controversial; however, this study suggests that IFMC could potentially enhance physical performance and advance the use of nanotechnology in sports. Since the detailed mechanism cannot be clarified based on the results of this experiment alone, the future research agendas are as follows: (1) investigations of various dependent variables related to cardiac function (heart rate, stroke volume, end-diastolic volume, end-systolic volume, and ejection fraction) as well as vascular response (flow mediated dilation, nitric oxide, red blood cell deformability and aggregation, blood lactate level, and oxygen transporting capacity parameters), (2) verification of the improvement in cardiopulmonary function by IFMC, particularly changes in blood oxygen levels and changes in percutaneous oxygen saturation during exercise in a hypoxic state, (3) clarification of the mechanism of NO expression by bringing IFMC into contact with or close to a living body, (4) investigation into the enhancement of other physical performance metrics (muscular endurance, instantaneous force, agility, or flexibility) through IFMC, (5) verification of improvement of autonomic balance (sympathetic nerve and parasympathetic nerve) through IFMC, and (6) investigation into other possibilities of IFMC. Multidisciplinary and/or trans-disciplinary studies will be required to explore these future areas of study.

## Figures and Tables

**Table 1:** Characteristics of the participants. The type of athlete includes (1) baseball, (2) ahletics, and (3) volleyball.

Subjects	1	2	3	4	5	6	7	8	9	10	11	12	13	14	15	16	17	18
Sex	M	M	M	M	M	M	M	M	M	F	F	F	F	F	F	F	F	F
Age	21	22	20	21	22	22	22	22	21	21	21	21	20	22	22	21	23	20
Height (cm)	175	174	183	169	173	159	173	169	162	162	161	168	158	165	165	171	168	166
Weight (kg)	87	72	86	62	60	63	63	68	54	F	45	65	49	66	F	62	56	58
Type of athlete	1	2	1	1	2	1	2	1	2	2	2	2	2	2	3	2	3	3
Smoking habits	No	No	No	No	No	No	No	No	No	No	No	No	No	No	No	No	No	No
Medication	No	No	No	No	No	No	No	No	No	No	No	No	No	No	No	No	No	No

## Data Availability

Original images and CSV files may be obtained from the corresponding author upon request.

## Abbreviations

The following abbreviations are used in this manuscript:

CO${}_{2}$	carbon dioxide
EDS	energy-dispersive X-ray spectrometer
FE-SEM	field emission scanning electron microscopy
Hb	hemoglobin
IFMC	Integrated Functional Mineral Crystal
NNI	National Nanotechnology Initiative
NO	nitric oxide
O	oxygen
